# Supplementary material for: Changes in Fatty Acid Dietary Profile Affect the Brain–Gut Axis Functions of Healthy Young Adult Rats in a Sex-Dependent Manner
Source: Nutrients. 2021 May 30;13(6):1864. doi: 10.3390/nu13061864 (PMC8228732; doi:10.3390/nu13061864)
Supplement: Supplementary file 1 [file nutrients-13-01864-s001.zip › nutrients-1186927-supplementary.pdf]

Supplementary material

Changes in fatty acid dietary profile affect the brain-gut axis functions of healthy young rats in a sex-dependent manner.

Damian Jacenik [1,a], Ana Bagüés [2,3,4,a], Laura López-Gómez [2,5], Yolanda López-Tofiño [2,5], Amaia Iriondo-DeHond [6], Cristina Serra [2], Laura Banovcanová [2], Carlos Gálvez-Robleño [2,5], Jakub Fichna [7], Dolores del Castillo [6], José A. Uranga [2,5] and Raquel Abalo [2,4,5,8*]

[1] Department of Cytobiochemistry, Faculty of Biology and Environmental Protection, University of Lodz, Lodz, Poland; damian.jacenik@biol.uni.lodz.pl
[2] Department of Basic Health Sciences, Faculty of Health Sciences, University Rey Juan Carlos (URJC), Alcorcón, Spain; ana.bagues@urjc.es; laura.lopez.gomez@urjc.es; yolanda.lopez@urjc.es; crisserra80@gmail.com; banovcanova.laura@gmail.com; carlos.galvezr@urjc.es; jose.uranga@urjc.es; raquel.abalo@urjc.es
[3] High Performance Research Group in Experimental Pharmacology (PHARMAKOM-URJC), URJC, Alcorcón, Spain; ana.bagues@urjc.es
[4] Associated I+D+i Unit to the Institute of Medicinal Chemistry (IQM), Scientific Research Superior Council (CSIC), Madrid, Spain; ana.bagues@urjc.es; raquel.abalo@urjc.es
[5] High Performance Research Group in Physiopathology and Pharmacology of the Digestive System (NeuGut-URJC), URJC, Alcorcón, Spain; laura.lopez.gomez@urjc.es; yolanda.lopez@urjc.es; carlos.galvezr@urjc.es; jose.uranga@urjc.es; raquel.abalo@urjc.es
[6] Food Bioscience Group, Department of Bioactivity and Food Analysis, Instituto de Investigación en Ciencias de la Alimentación (CIAL) (CSIC-UAM), Calle Nicolás Cabrera, 9, 28049 Madrid, Spain; amaia.iriondo@csic.es; mdolores.delcastillo@csic.es
[7] Department of Biochemistry, Faculty of Medicine, Medical University of Lodz, Lodz, Poland; jakub.fichna@umed.lodz.pl
[8] Working Group of Basic Sciences in Pain and Analgesia of the Spanish Pain Society (Grupo de Trabajo de Ciencias Básicas en Dolor y Analgesia de la Sociedad Española del Dolor), Madrid, Spain
[a] Same contribution
* Correspondence: raquel.abalo@urjc.es; Tel.: +34-91-488-88-54

Table S1. Composition in fatty acids of soybean oil, coconut oil and evening primrose oil.

Type of fatty acid	Soybean oil (%)	Coconut oil (%)	Evening primrose oil (%)
**SATURATED FAs**	**14**	**90.4**	**8.2**
C8:0 caprylic acid		7.1	
C10:0 capric acid		5.7	
C12:0 lauric acid		46.8	
C14:0 myristic acid		18.2	
C16:0 palmitic acid	10	9.8	6.3
C18:0 stearic acid	4	2.8	1.9
**MONOUNSATURATED LCFAs**	**23**	**6.8**	**6.9**
C18:1 n-9 oleic acid	23	6.8	6.9
**POLYUNSATURATED LCFAs**	**58**	**2**	**83.1**
C18:2 n-6 linoleic acid	51	1.8	73.9
C18:3 n-3 linolenic acid	7	0.1	
C18:3 n-6 gamma-linolenic acid		0.1	9.2
**Others**	**5**	**0.8**	**1.8**
Total	100		

Abbreviations: FAs – fatty acids; LCFAs – long-chain fatty acids. Note: FAs are classified as saturated FAs, monounsaturated FAs and polyunsaturated FAs, according to the presence and number of double bounds in their molecular structure. Another classification of FAs is based on number of carbons, where FAs are divided into short-, medium-, or long-chain FAs. Short-chain FAs contain fewer than 6 carbons, medium-chain fatty acids contain 6 to 12 carbons and LCFAs contain more than 12 carbons.

Table S2. Body weight and macroscopic characteristics of fat and gastrointestinal organs at sacrifice.

		Sex	SOY	COCO	EP
**Weight of animals (g)**		Males	378.33±8.34	384.50±20.72	446.00±7.52**
		Females	245.33±5.83	260.33±12.5	240.17±4.44
**Weight of fat (% of body weight)**		Males	7.30±0.90	7.35±1.00	10.60±1.10
		Females	8.24±0.70	8.34±1.10	6.89±0.40
**Weight (% of body weight)**	**Stomach**	Males	1.45±0.74	1.11±0.05	0.90±0.27
		Females	1.00±0.25	1.14±0.32	1.45±0.37*
	**Full SI**	Males	1.79±0.1	1.93±0.14	1.82±0.17
		Females	2.27±0.14	2.40±0.15	2.26±0.21
	**Empty SI**	Males	1.72±0.46	1.82±0.59	1.55±0.09
		Females	1.98±0.11	2.01±0.12	1.93±0.14
	**Milking**	Males	0.25±0.07	0.30±0.06	0.24±0.03
		Females	0.21±0.07	0.32±0.07	0.31±0.09
	**Cecum**	Males	0.66±0.08	0.60±0.14	0.57±0.08
		Females	0.93±0.2	1.06±0.15	0.79±0.1
	**Full colorectum**	Males	0.54±0.09	0.54±0.07	0.58±0.07
		Females	0.76±0.27	0.69±0.19	0.62±0.08
	**Empty colorectum**	Males	0.36±0.04	0.40±0.06	0.35±0.03
		Females	0.51±0.06	0.45±0.04	0.54±0.07
**Area or length**	**Stomach (cm²)**	Males	5.02±1.31	4.70±0.57	4.71±0.90
		Females	3.33±0.38	3.73±0.50	4.15±0.86
	**Cecum (cm²)**	Males	4.31±0.41	3.85±0.43	4.00±0.56
		Females	3.66±0.47	3.95±0.52	2.93±0.49*
	**SI (cm)**	Males	51.50±4.00	49.20±3.78	56.00±7.17
		Females	49.80±2.58	49.40±2.70	47.90±5.74
	**Colorectum (cm)**	Males	8.75±0.56	9.35±1.03	10.08±0.63
		Females	9.82±1.35	8.97±0.70	7.75±0.49**

Male and female rats were exposed to AIN-93G diet containing 7% of soybean oil (SOY), or to modified AIN-93G diet containing 3.5% of soybean oil supplemented with 3.5% of coconut oil (COCO) or with 3.5% of evening primrose oil (EP). Data were analyzed for males and females separately. Data are expressed as the means ± S.D. (n = 6 animals per group). *p < 0.05, **p < 0.01 vs. SOY males or females. One-way ANOVA followed by Bonferroni´s multiple comparison test. Abbreviations: SI, small intestine.

Table S3. Histological quantification of adipose cell area.

	Sex	SOY	COCO	EP
**Adipocyte area (µm² x 10³)**	Males	2.84 ± 0.81	3.60 ± 1.51	3.70 ± 2.36
	Females	3.17± 1.45	3.52± 1.56	2.96 ± 0.82

Effects of diet intervention on adipose cell area in the rat. Male and female rats were exposed to AIN-93G diet containing 7% of soybean oil (SOY), or to modified AIN-93G diet containing 3.5% of soybean oil supplemented with 3.5% of coconut oil (COCO) or with 3.5% of evening primrose oil (EP). Data were analyzed for males and females separately. Data are expressed as the means ± S.D. (n = 6 animals per group). One-way ANOVA followed by Bonferroni´s multiple comparison test.

GENERAL HEALTH PARAMETERS

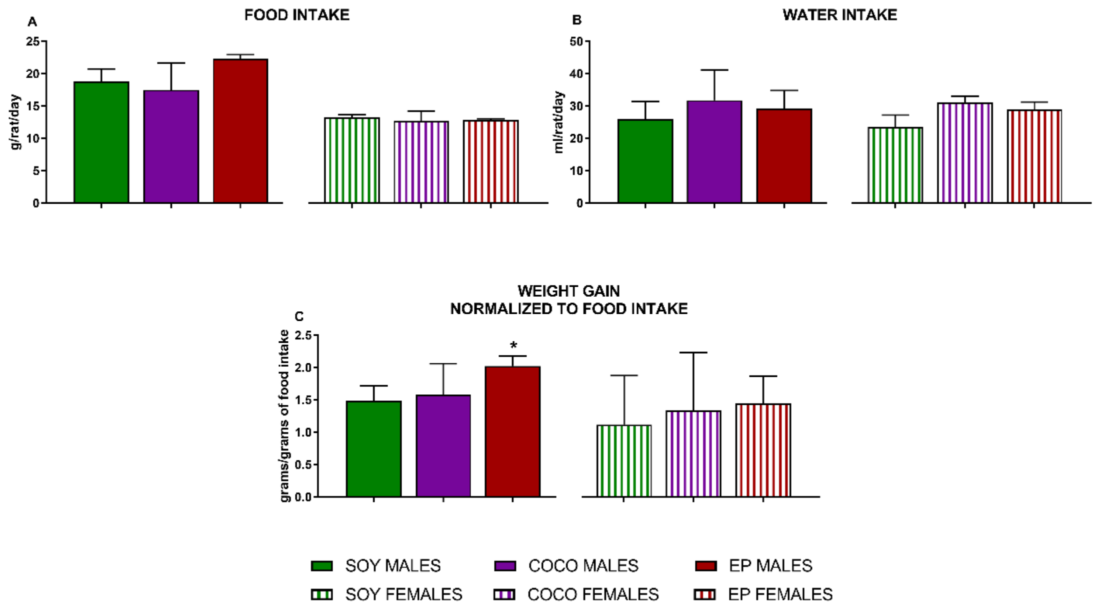

Figure S1. Effects of diet intervention on general health parameters in male and female rats. Animals were exposed to AIN-93G diet containing 7% of soybean oil (SOY), or to modified AIN-93G diet containing 3.5% of soybean oil supplemented with 3.5% of coconut oil (COCO) or with 3.5% of evening primrose oil (EP). Food (A) and water (B) intakes, and body weight gain normalized to food intake (C) during the six weeks of diet exposure were recorded and analyzed for the three male groups and the three female groups separately. Data are expressed as mean ± S.D. (n = 6-12 animals per group). One-way ANOVA followed by Bonferroni's multiple comparison test. *$p < 0.05$ vs SOY males.

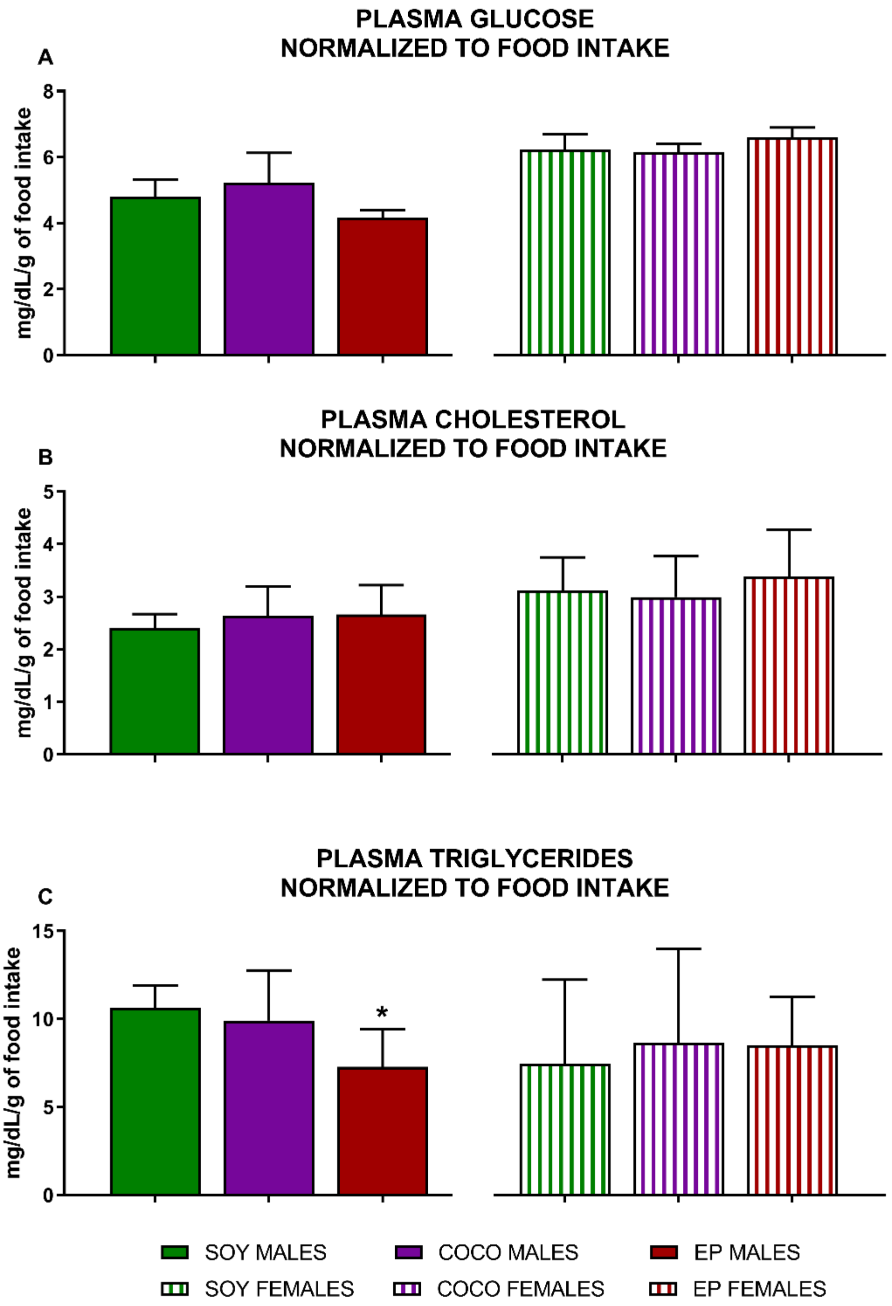

Figure S2. Effects of diet intervention on plasma levels of glucose and lipids in male and female rats. Animals were exposed to AIN-93G diet containing 7% of soybean oil (SOY), or to modified AIN-93G diet containing 3.5% of soybean oil supplemented with 3.5% of coconut oil (COCO) or with 3.5% of evening primrose oil (EP). Random plasma glucose (A), cholesterol (B) and triglyceride (C) levels after normalization to food intake were analyzed for the three male groups and the three female groups separately. Data are expressed as mean ± S.D. (n = 6 animals per group). One-way ANOVA followed by Bonferroni's multiple comparison test. *$p < 0.05$ vs SOY males.

ADIPOCYTE SIZE

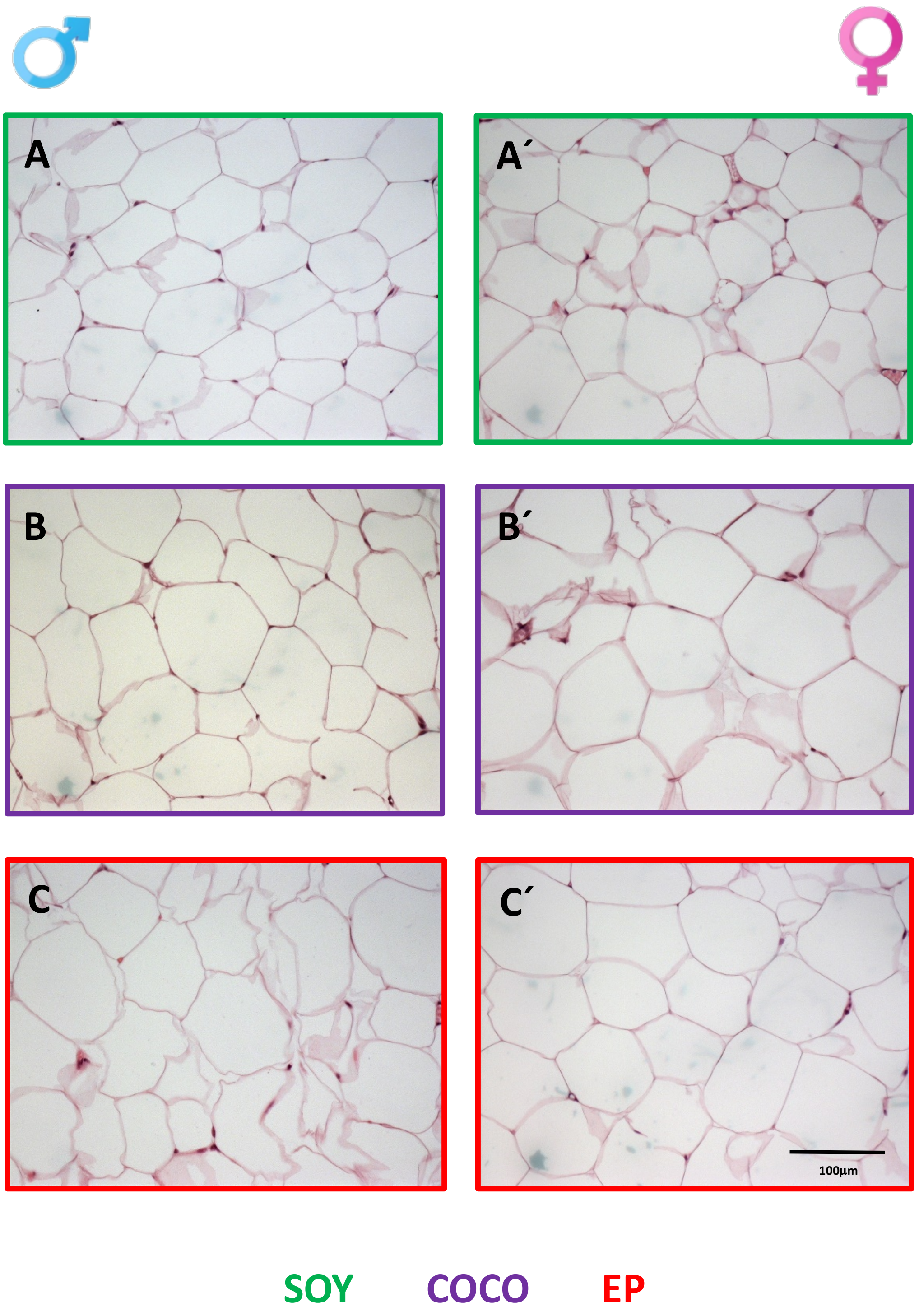

Figure S3. Representative images of hematoxylin and eosin staining of adipose tissue obtained from male (A, B, C) and female (A′, B′, C′) rats under AIN-93G diet containing 7% of soybean oil (SOY, A, A′), AIN-93G diet containing 3.5% of soybean oil supplemented with 3.5% of coconut oil (COCO, B, B′) or 3.5% of evening primrose oil (EP, C, C′). Scale bar: 100 µm.

ILEUM STRUCTURE

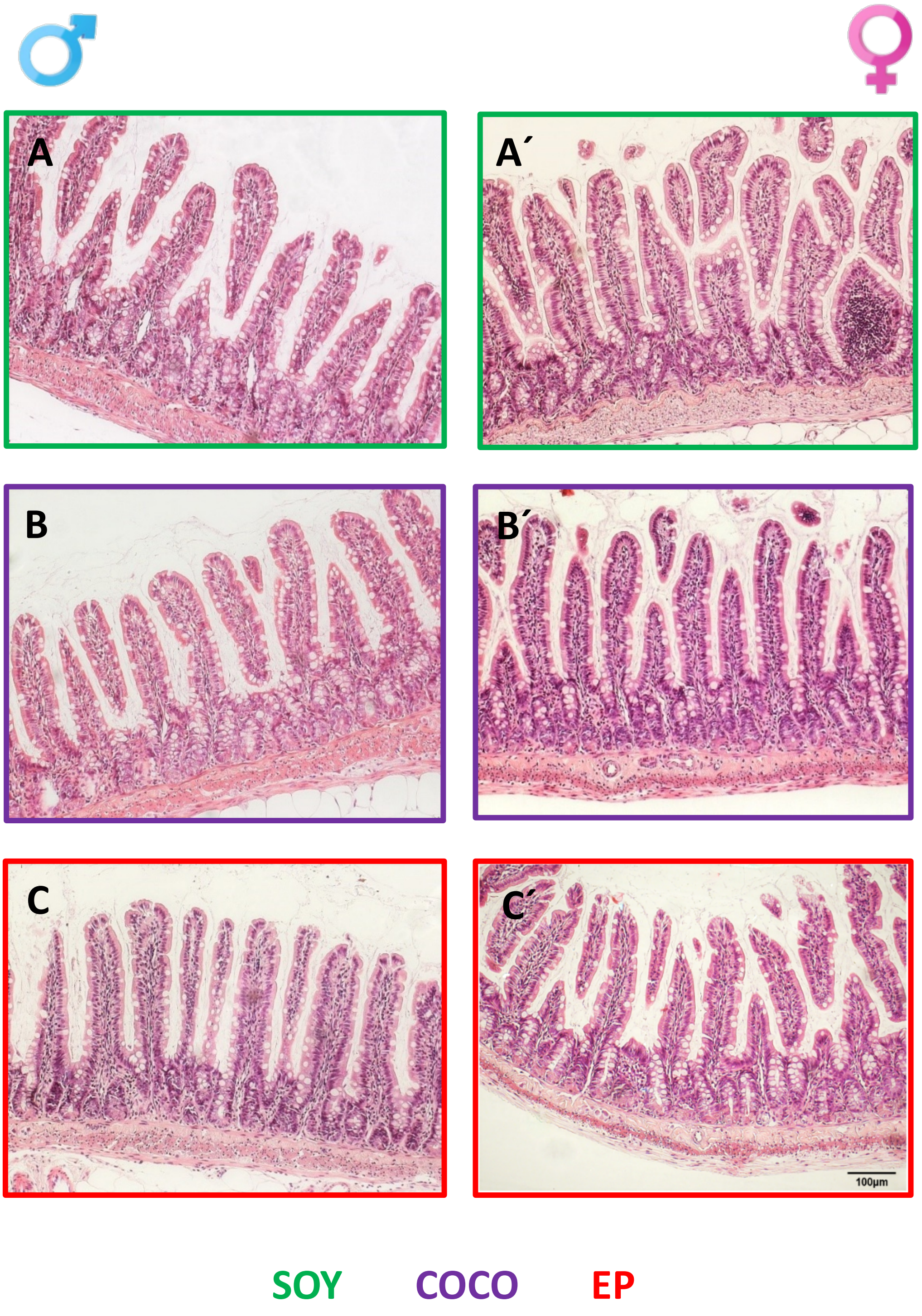

Figure S4. Representative images of hematoxylin and eosin staining of ileum obtained from male (A, B, C) and female (A', B', C') rats under AIN-93G diet containing 7% of soybean oil (SOY, A, A'), AIN-93G diet containing 3.5% of soybean oil supplemented with 3.5% of coconut oil (COCO, B, B') or 3.5% of evening primrose oil (EP, C, C'). Scale bar: 100 µm.

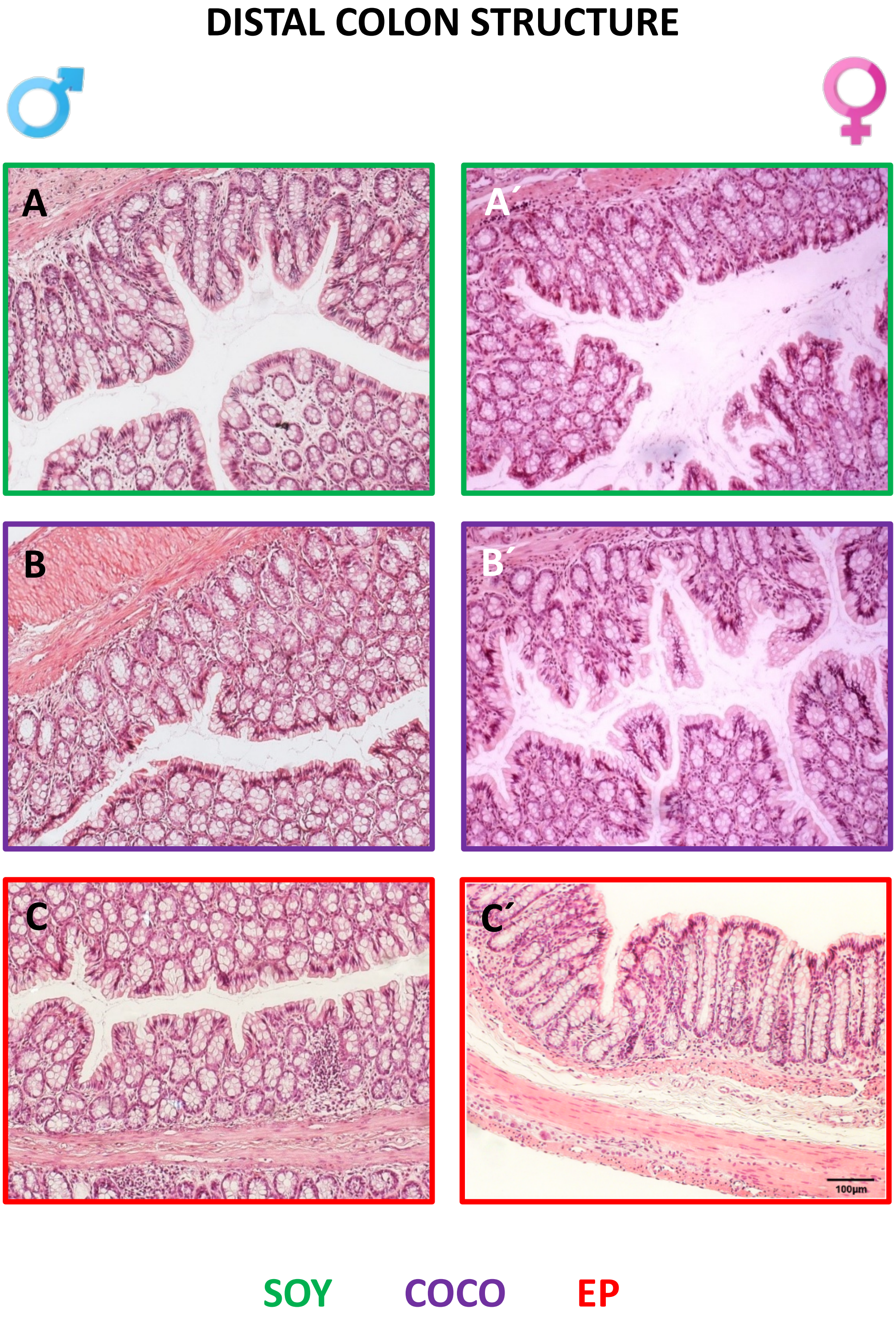

Figure S5. Representative images of hematoxylin and eosin staining of colon obtained from male (A, B, C) and female (A', B', C') rats under AIN-93G diet containing 7% of soybean oil (SOY, A, A'), AIN93G diet containing 3.5% of soybean oil supplemented with 3.5% of coconut oil (COCO, B, B') or 3.5% of evening primrose oil (EP, C, C'). Scale bar: 100 μm.

MAST CELL INFILTRATION-DISTAL COLON

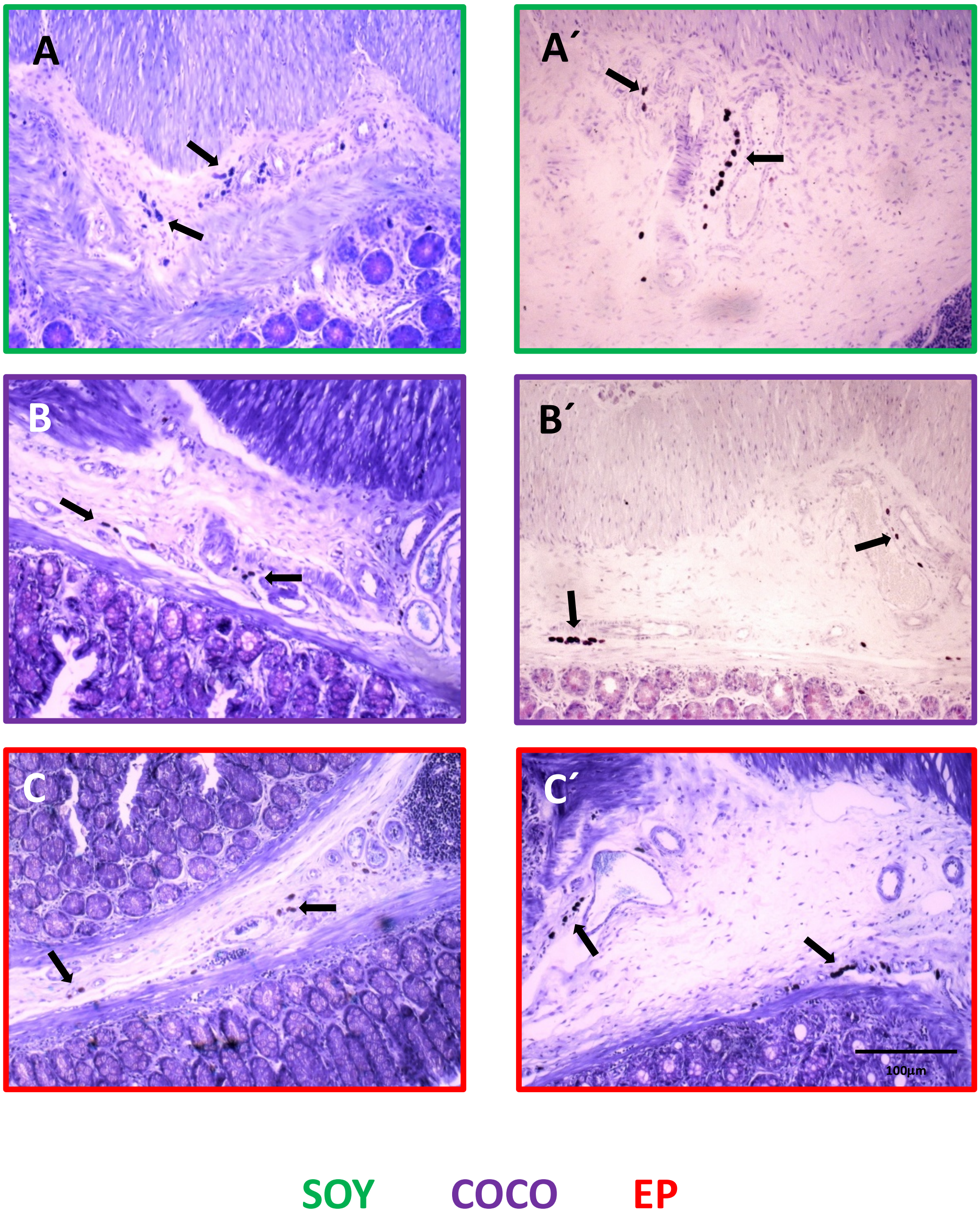

Figure S6. Representative images of toluidine blue staining of colon obtained from male (A, B, C) and female (A', B', C') rats under AIN-93G diet containing 7% of soybean oil (SOY, A, A'), AIN93G diet containing 3.5% of soybean oil supplemented with 3.5% of coconut oil (COCO, B, B') or 3.5% of evening primrose oil (EP, C, C'). Scale bar: 100 µm.
